# Loss of fiber cell communication may contribute to the development of cataracts of many different etiologies

**DOI:** 10.3389/fphys.2022.989524

**Published:** 2022-09-12

**Authors:** Eric C. Beyer, Richard T. Mathias, Viviana M. Berthoud

**Affiliations:** ^1^ Department of Pediatrics, University of Chicago, Chicago, IL, United States; ^2^ Department of Physiology and Biophysics, Stony Brook University, Stony Brook, NY, United States

**Keywords:** connexin, gap junction, lens, cataract, cell-to-cell communication

## Abstract

The lens is an avascular organ that is supported by an internal circulation of water and solutes. This circulation is driven by ion pumps, channels and transporters in epithelial cells and by ion channels in fiber cells and is maintained by fiber-fiber and fiber-epithelial cell communication. Gap junctional intercellular channels formed of connexin46 and connexin50 are critical components of this circulation as demonstrated by studies of connexin null mice and connexin mutant mice. Moreover, connexin mutants are one of the most common causes of autosomal dominant congenital cataracts. However, alterations of the lens circulation and coupling between lens fiber cells are much more prevalent, beyond the connexin mutant lenses. Intercellular coupling and levels of connexins are decreased with aging. Gap junction-mediated intercellular communication decreases in mice expressing mutant forms of several different lens proteins and in some mouse models of lens protein damage. These observations suggest that disruption of ionic homeostasis due to reduction of the lens circulation is a common component of the development of many different types of cataracts. The decrease in the lens circulation often reflects low levels of lens fiber cell connexins and/or functional gap junction channels.

## Introduction: The lens

The lens is an avascular organ that is suspended near the front of the eye between the aqueous and vitreous humors. It is a normally transparent organ that facilitates the focusing of light onto the retina. Damage to cells within the lens and their contents can lead to opacities or cataracts that scatter light or impede its transmission. Therefore, physiological processes that support the health of lens cells are critical for the maintenance of normal vision.

## The lens circulation facilitates the maintenance of transparency

**FIGURE 1:**
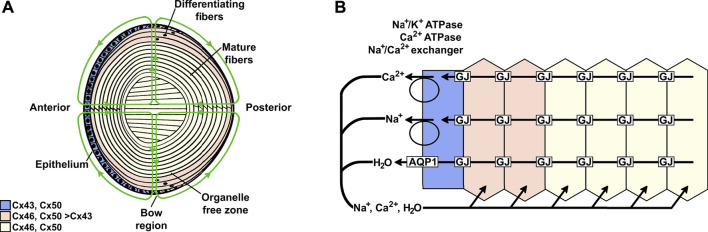
Model of the lens and the distribution of different connexins. **(A)** The lens contains two cell types: an anterior epithelial cell layer and elongated fiber cells that constitute the bulk of the organ. At the lens equator (bow region), the cells start differentiating, a process that includes elongation of the cells and the loss of organelles to form fiber cells that are added as sequential layers. Thus, the differentiating fiber cells are more superficial, and the mature fibers are located deeper within the lens. Three connexins (Cx43, Cx46, and Cx50) are differentially distributed in the regions of the lens. The green lines indicate the lens circulation of water and ions; arrowheads indicate the direction of flow. The movement of fluid is coupled to the circulation of ions. **(B)** Diagram illustrating the movement of Na^+^, Ca^2+^, and water in a cellular column from the lens. Ions enter into the lens through the spaces between cells at the anterior and posterior poles and move towards the lens center. As they move to the center, ions are driven into fiber cells by their electrochemical gradients; then, they flow from cell-to-cell from the lens center to the epithelial cells on the lens surface through gap junction channels and exit the lens at the bow region. The ions are transported out of the lens by epithelial pumps, and water moves out through AQP1 channels.

The lens comprises an anterior epithelial cell layer and elongated fiber cells that constitute the bulk of the organ (Figure 1). All of the lens nutrients derive from the ocular humors and are delivered to fiber cells throughout the volume by the lens circulation (green lines of flow in Figure 1). The circulation is ultimately driven by the activity of the Na^+^/K^+^ ATPase, which is concentrated in equatorial surface cell membranes (Garner and Kong, 1999; Gao et al., 2000; Candia and Zamudio, 2002; Tamiya et al., 2003). The ATPase generates a transmembrane electrochemical gradient for sodium to enter all fiber cells. Sodium entry into fiber cells creates transmembrane osmotic gradients that cause fluid to follow. Sodium and fluid flow into the lens through spaces between cells, where the fluid flow convects nutrients and antioxidants from the humors to fiber cells throughout the lens volume. These solutes are taken up by fiber cell membrane transporters and used for intracellular anaerobic metabolism and oxidative protection. The sodium that has entered fiber cells flows through gap junctions to the lens surface, where it is extruded by the Na^+^/K^+^ ATPase. Again, fluid follows. Intracellular waste byproducts are convected through gap junctions to lens surface cells for breakdown and metabolism and/or extrusion back into the humors (reviewed in Mathias et al., 2007).

### Connexin mutations disrupt the lens circulation and the maintenance of transparency

**FIGURE 2:**
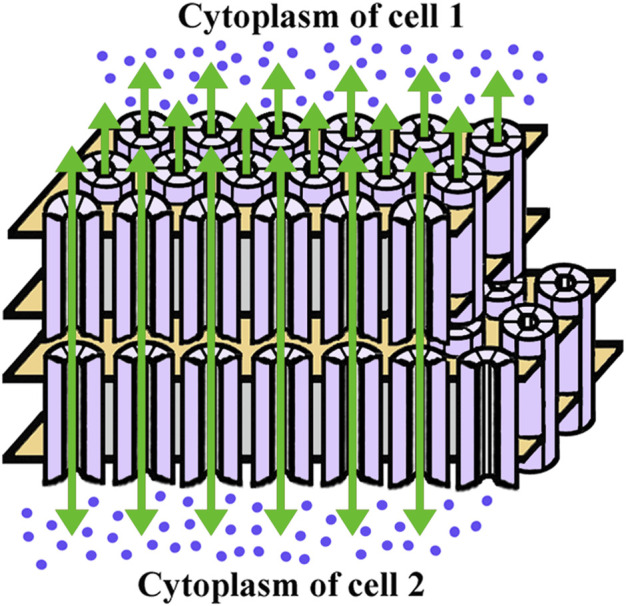
Diagram of the gap junction structure. A gap junction is a cluster of intercellular channels formed by head-to-head apposition of connexin hemichannels within the plasma membranes of adjacent cells. The hemichannels are hexameric assemblies of connexin proteins surrounding a central aqueous pore and are depicted as cylinders formed of six subunits (light violet). The boundaries of the plasma membrane are illustrated in gold. The green arrows indicate the intercellular passage of ions and small molecules (ultramarine blue dots) through the gap junction channels. While the channels allow bidirectional flow, there is a net outwards flux of water and ions in the lens.

Within the lens circulation system, lens fiber cell gap junctions provide the intracellular outflow pathway that allows ions and molecules to cross cell boundaries without being released to the extracellular spaces. Gap junctions are plasma membrane specializations that contain clusters of cell-to-cell channels formed by subunit proteins called connexins (Figure 2). Three different connexin subtypes are expressed in the lens. Connexin43 (Cx43) is expressed in epithelial and differentiating cells, connexin46 (Cx46) is expressed in differentiating and mature fiber cells, and connexin50 (Cx50) is expressed in epithelial cells and differentiating and mature fiber cells (Figure 1) (Beyer et al., 1989; Musil et al., 1990; Paul et al., 1991; White et al., 1992; Berthoud et al., 1994; Jiang et al., 1994; Rong et al., 2002).

The permeability of gap junction channels to water, ions and small molecules (including many nutrients and metabolites) suggests that the maintenance of lens transparency depends on gap junction-mediated cell-to-cell communication (Goodenough, 1992; Mathias et al., 2010). This hypothesis has been supported by multiple lines of evidence. Mutations of the genes encoding Cx46 or Cx50 (*Gja3* or *Gja8*) are linked to the development of congenital cataracts in people. As of the current time, 179 alterations of these genes (leading to changes in 57 different amino acids in Cx50 and 42 in Cx46) have been identified and associated with cataracts (https://cat-map.wustl.edu/). Some of the connexin mutations identified in members of families with inherited cataracts are reviewed in Beyer et al. (2013) and Berthoud and Ngezahayo (2017). Cataracts also develop in homozygous Cx46-and Cx50-null mice (Gong et al., 1997; White et al., 1998; Rong et al., 2002) and in mice carrying Cx46 or Cx50 gene mutations (Steele et al., 1998; Graw et al., 2001; Chang et al., 2002; DeRosa et al., 2009; Xia et al., 2012; Berthoud et al., 2013; Berthoud et al., 2014).

Several studies have demonstrated that mutations in either of the lens fiber cell connexins, Cx46 or Cx50, result in decreased intercellular communication between fiber cells, and, consequently, disruptions of the lens circulation and ion homeostasis (Gong et al., 1998; Baldo et al., 2001; Martinez-Wittinghan et al., 2004; Gao et al., 2011; Gao et al., 2018; Minogue et al., 2017; Berthoud et al., 2019). Decreased intercellular coupling also occurs in animals with heterozygous deletion of Cx46 or Cx50 (Gong et al., 1998; Baldo et al., 2001). Interestingly, in the heterozygous Cx50 knockout the coupling conductance decreases only in the differentiating fibers, but it is normal in the mature fibers (Baldo et al., 2001), whereas in heterozygous Cx46 knockout mice, the coupling conductance decreases in both differentiating and mature fibers (Gong et al., 1998).

## Reductions in levels of fiber cell connexins and/or gap junction-mediated intercellular communication are associated with cataracts from other causes

Reductions of connexins and gap junction-mediated communication between lens fiber cells also occur in mouse models that do not result from direct manipulations or abnormalities of the lens fiber connexin genes. In many cases, these changes occur early enough to suggest that they contribute to cataract pathogenesis. The lenses of these mice have disruptions of the lens internal circulation and various ionic imbalances (summarized in Table 1 and described in more detail below).

**TABLE 1:** Mouse models with alterations of fiber cell gap junction-mediated intercellular communication (GJIC) and/or levels of fiber cell connexins.

Mutant	GJIC	Lens fiber connexin level	Hydrostatic pressure	[Na^+^]	[Ca^2+^]	Reference(s)
Aging wild type	↓	↓	↑	↑	↑	Gao et al. (2013)
Lim2 knockout	↓	↓ Cx46 (lens nucleus)				Shiels et al. (2007b), Shi et al. (2011)
GPX-1 knockout	↓	↓	↑	↑	↑	Wang et al. (2009); Gao et al. (2011)
CRYGB-S11R		↓			↑	Li et al. (2010)
K6W-Ub	↓	↓			↑	Liu et al. (2015)

### Aging mice

Studies of lenses from wild-type mice showed that gap junctional coupling conductance decreases with increasing age (Gao et al., 2013). This change is associated with decreases in levels of the lens fiber cell connexins. In these mice, the gradients of hydrostatic pressure and the intracellular concentrations of Na^+^ and Ca^2+^ increase between 2 and 14 months, likely due to impairment of the ionic outflow pathway (the component of the lens circulation that is normally provided by Cx46 and Cx50 gap junctions). In this study, no significant opacities were detected in the wild-type mice at 14 months of age (Gao et al., 2013). The absence of cataracts implies that wild-type lenses are capable of developing some compensatory mechanisms to prevent their formation. However, the reductions of fiber cell connexins and fiber cell communication associated with changes in the lens circulation and ionic imbalances likely contribute to the increased susceptibility of the aging lens to cataract formation.

### *Lim2* gene-trap lenses

The function of lens intrinsic membrane protein-2 (LIM2) in the lens was studied by generating mice with *Lim2* null-alleles using a gene-trap approach (*Lim2* ^*Gt/Gt*^). Homozygous *Lim2* ^*Gt/Gt*^ lenses develop faint, central pulverulent cataracts (Shiels et al., 2007b). In these lenses, the lens fiber membrane potential is slightly (but significantly) depolarized, and gap junctional coupling is decreased by 55% in the differentiating fibers and in the mature fibers. The decrease in gap junctional coupling is associated with a ∼50% decrease in the protein levels of Cx46 in mature fibers (but levels are similar to wild-type lenses in the cortical differentiating fiber region) (Shi et al., 2011).

These lenses may also have reductions of the lens circulation due to non-connexin mechanisms. The *Lim2* ^*Gt/Gt*^ lenses lack cellular fusions. Because levels of Cx46 are similar to wild-type lenses in the cortical differentiating fiber region, the authors attribute the decrease in intercellular coupling in the cortical region to the absence of cellular fusions (Shi et al., 2011).

### Glutathione peroxidase-1 knockout lenses

Glutathione peroxidase-1 (GPX-1) is the most abundant isozyme of the glutathione peroxidase family. It catalyzes the reduction of peroxides by glutathione, and thereby provides protection to the lens from oxidative damage. A recent study showed that GPX-1 knockout lenses, but not catalase knockout lenses, showed accelerated abnormal optical aberrations and cataracts with increasing age (Varadaraj et al., 2022), suggesting that GPX-1 provides primary protection for the lens against oxidative damage. Although GPX-1 knockout lenses are transparent early in life, they eventually develop nuclear cataracts (Reddy et al., 2001; Varadaraj et al., 2022). At 2 months of age, when lenses are clear, GPX-1 knockout lenses show decreases in coupling conductance to 72% of wild-type values in the differentiating fibers and to 45% of wild-type values in the mature fibers. These changes are associated with an accumulation of Ca^2+^ and Na^+^ in the lens nucleus, a ∼50% decrease in Cx46 and Cx50 levels, and an increase in the gradient of hydrostatic pressure (Wang et al., 2009; Gao et al., 2011).

### *Crygb^S11R^* lenses

Homozygous mice carrying the S11R mutation of γ_B_-crystallin (*Crygb^S11R^*) progressively develop severe cataracts (Li et al., 2008). The lenses of these mice appear free of opacities at post-natal day 1, contain detectable opacities at post-natal day 7, and have dense nuclear cataracts by the age of weaning. While no changes in connexin levels were detected in these lenses at post-natal day 1, they showed a ∼4-fold decrease in Cx46 and a ∼5-fold reduction in Cx50 at post-natal day 10. In the homozygous mutant lenses, the level of total lens Ca^2+^ was four times the level in wild-type lenses. These lenses have a variety of biochemical and structural abnormalities including aggregation of γ-crystallins adjacent to the cell boundary of inner mature fiber cells, cleaved crystallins, disruption of actin filaments, and degeneration of inner fiber cells (Li et al., 2008). The importance of altered fiber cell gap junctions in the pathogenesis of these cataracts was suggested by studies of *Crygb^S11R^* mice in which Cx46 was “knocked-in” into the Cx50 locus (Li et al., 2010). This intervention resulted in clear lenses without histopathology that had a normal distribution of filamentous actin and cytosolic γ-crystallin in interior fiber cells. It also prevented the increase in Ca^2+^ levels and degradation of crystallins (although lenses were smaller in size due to the absence of Cx50).

### Transgenic mice overexpressing ubiquitinK6W

Transgenic mice expressing ubiquitinK6W (a mutant ubiquitin that inhibits ubiquitin-dependent protein degradation) develop nuclear cataracts (Liu et al., 2015). Opacities are visible at post-natal day 1. The ubiquitinK6W mice have a ∼3-fold increase in the gap junction series resistance (i.e., reduced intercellular coupling) in the lens nucleus as compared with wild-type mice; however, this parameter is not altered at the lens surface. Some ionic imbalances may be consequences of the increased gap junction series resistance. The mutant mice have >4.5-fold elevation in the Ca^2+^ concentration in the nucleus of the lens, which is a little higher than the level that would be predicted from the increase of the series resistance in this region. These mice also have a 4-fold increase in the Ca^2+^ concentration in the lens cortical area (even though the series resistance in this region is not different from that of wild-type lenses).

Alteration in the levels of lens connexins may partially explain the impairment of the lens circulation in these mutant mice. Mass spectrometry studies revealed 1.8-fold and 1.5-fold decreases in levels of Cx46 and Cx50, respectively, in the ubiquitinK6W lenses. The decreases in Cx46 and Cx50 levels may result from activation of the Ca^2+^-dependent protease, calpain, whose activity was shown to be increased in these lenses (Liu et al., 2015). Although Cx43 was not detected in the mass spectra, immunoblotting showed that the level of Cx43 was increased ∼1.8-fold in the ubiquitinK6W lenses. The ubiquitinK6W lenses also had more ubiquitinated Cx43 conjugates than wild-type lenses. Determination of mRNA levels showed that the changes in levels of Cx43 and Cx46 did not result from alterations in their transcript levels. Therefore, it is likely that the increases in Cx43 and ubiquitinated Cx43 result from impaired degradation, since conjugates containing ubiquitin K6W are resistant to proteasomal degradation.

### Possible mechanisms accounting for the decreases in fiber cell connexins and intercellular channels

The steady-state levels of the lens fiber cell connexins depend on the balance between their synthesis and degradation. Therefore, the reduced levels in these different cataract models must reflect alterations of one or both of these processes.

Formation of fiber cell gap junction plaques occurs in the outer layer of differentiating fibers, where it involves transcription of connexin mRNAs, translation of the connexin polypeptides, cellular trafficking, assembly of the channels and clustering into gap junction plaques. In some cases, levels of fiber cell connexin mRNAs have been determined and are not altered (Liu et al., 2015); but, in many models, they have not been studied. However, it is likely that transcription of Cx46 (and possibly Cx50) mRNA would be reduced in lenses in which differentiation of epithelial cells into fiber cells is impaired. Levels of connexin proteins might be reduced in models in which endoplasmic reticulum stress is activated leading to decreased translation of most proteins, except those involved in counteracting the stress (like chaperones). Mutations, diseases, or treatments that affect proteins required for connexin targeting to the plasma membrane or that are required for their function at the plasma membrane would lead to decreases in gap junction channel function and/or plaques. Incompletely trafficked/assembled connexins (that never reached the plasma membrane) would likely be recognized by the “quality control” system and targeted for degradation.

Connexins are subject to degradation by the proteasome and the lysosome. Degradation of connexins can only occur in cells that contain the appropriate machinery. Since fiber cells lose their organelles during differentiation, we infer that connexin degradation through these pathways occurs in epithelial and differentiating fiber cells, but not mature fiber cells in wild-type lenses. The proteasome is the major site for degradation of newly synthesized connexin polypeptides that have not reached the plasma membrane. This process includes the removal of mis-folded and mis-assembled proteins by the “quality control” system. In the case of the ubiquitinK6W overexpressing lenses, no proteasomal degradation of Cx46 or Cx50 should occur if the attached polyubiquitin chains contain the mutant ubiquitinK6W. Lysosomal degradation is the primary pathway for fiber cell connexins contained within channels and gap junctions that have been internalized from the plasma membrane although some connexin may be directly transported to the lysosome from early secretory compartments (Qin et al., 2003; Minogue et al., 2022); degradation of internalized/cytoplasmic connexin assemblies may involve autophagy (Lichtenstein et al., 2011). Lysosomal or autophagosomal degradation of connexins has not been examined in any of the animal models discussed above.

Specific proteases might contribute to the reductions of connexin levels. Degradation of connexins by members of the family of Ca^2+^-dependent proteolytic enzymes, the calpains, has been invoked in several cataract models (Nakamura et al., 2000; Tang et al., 2007; Liu et al., 2015). However, calpain activity may not fully explain loss of fiber cell connexins, because calpains only cause fragmentation/truncation of connexins (Kistler and Bullivant, 1987; Lin et al., 1997), and constructs corresponding to some (but not all) of the truncated Cx46 and Cx50 products retain channel function in expression systems (Lin et al., 1998; Xu et al., 2002; DeRosa et al., 2006; Slavi et al., 2016). Caspases have also been implicated in cleaving connexins (Yin et al., 2001; Wang et al., 2012). Some of the truncated forms of Cx46 and Cx50 identified in human lenses may result from non-enzymatic cleavage of the connexins similar to that described for crystallins and AQP0 (Voorter et al., 1988; Ball et al., 2004; Lyons et al., 2016; Slavi et al., 2016). Thus, the decrease in connexin levels in these mouse models might result from sequential steps: cleavage of the connexin (non-enzymatic, by caspases or by calpain if the concentration of intracellular calcium ions is sufficiently high to activate the enzyme), followed by complete degradation of the connexin fragments in the proteasome or the lysosome. The extent of truncation/degradation of connexins and gap junctions likely increases with age. Indeed, Cx45.6 (the chicken ortholog of Cx50) undergoes a development-associated truncation (Yin et al., 2001). The abundance of truncations within the N-terminal region and the cytoplasmic loop of Cx46 and Cx50 in human lenses increases dramatically from outer cortex to nucleus (Slavi et al., 2016). In addition, lens proteins (including the connexins) accumulate other forms of modifications and damage with progressive aging. This may be the reason why the loss of coupling conductance is much greater in the mature fibers than in the differentiating fibers in some models.

## Some mouse models cause alterations of fiber cell gap junctions without necessarily altering connexin levels

Lens fiber gap junctions and the function of the cell-to-cell channels that they contain can be disrupted as a consequence of other cellular changes like alterations of cytoskeletal or membrane proteins that disturb plasma membrane organization.

### Tmod1 and CP49 double knockout lenses

Simultaneous deletion of Tmod1 (an actin pointed-end capping protein) and CP49 (a lens-specific intermediate filament protein) disrupts cytoskeletal networks in lens fiber cells. These lenses show increased light scattering, but not overt cataracts (at least through 4 months of age) (Gokhin et al., 2012). The loss of Tmod1 and CP49 causes an abnormal “moth-eaten” appearance of the actin-spectrin network, patches of disorganized and misaligned fiber cells in the outer cortex of the lens and a decrease in lens stiffness (Gokhin et al., 2012). These lenses show increased gap junction coupling resistance (i.e., lower intercellular communication), hydrostatic pressure, and sodium concentration. These increases are not associated with changes in Cx46 or Cx50 levels; however, Cx46 gap junction plaques are smaller and more dispersed in differentiating fiber cells while the localization and sizes of Cx50 gap junction plaques are unaffected. These results imply differential influences of the cytoskeleton on accretion of different connexins into large gap junction plaque domains or on the stability of large plaques made of Cx46 or Cx50 (Cheng et al., 2015).

### AQP0-null and AQP0-mutant lenses

Aquaporin-0 (AQP0; also known as major intrinsic protein, MIP) is the most abundant membrane protein in the lens. Like other members of the aquaporin family, AQP0 is a water channel (Mulders et al., 1995; Kushmerick et al., 1998; Varadaraj et al., 2005). AQP0 also has been implicated in adhesion between fiber cells (Kumari and Varadaraj, 2009; Froger et al., 2010). There are several mutations in AQP0 that result in congenital, progressive or age-related cataracts in people (for a compilation, see Cat-Map https://cat-map.wustl.edu/). In mice, a mutation known as *Cat* ^*Fr*^ and as AQP0-LTR results in the replacement of the C-terminus of AQP0 with a long terminal repeat (LTR) such that amino acids 203–261 are different in AQP0-LTR (Shiels and Bassnett, 1996). AQP0-LTR fails to traffic to the membrane and, by 3 weeks of age, homozygous AQP0-LTR mice have bilateral cataracts with an anterior sub-polar opacity (Shiels et al., 2000). Fiber cells within the nucleus of these mice are severely disordered. The levels of fiber cell connexins and the distribution and function of gap junctions have not been assessed in these mice, but they would be expected to be abnormal (at least in the homozygotes). Fiber cell-to-fiber cell gap junction coupling conductance could not be measured in homozygous *Cat* ^*Fr*^ lenses (which are smaller and opaque), but no changes in gap junction coupling or conductance were found between wild-type and heterozygous *Cat* ^*Fr*^ lenses (Varadaraj et al., 1999). However, the fiber cell membrane water permeability (*p*_*H2O*_) was reduced by 13 μm/s in heterozygotes and by 30 μm/s in homozygotes compared to the value of 43 μm/s in wild-type fiber cell membranes (Varadaraj et al., 1999).

AQP0-null mice have also been generated and studied (Shiels et al., 2001). Lenses from homozygous AQP0-null mice develop polymorphic opacities by postnatal day 21, while the lenses from heterozygous AQP0-null mice do not develop frank opacities until approximately 24 weeks of age. The lenses of homozygous AQP0-null mice show significant abnormalities in the fiber cell structure, and they fail to form sutures. In addition, the size and abundance of gap junction plaques are significantly decreased in both heterozygous and homozygous AQP0-null lenses (Al-Ghoul et al., 2003). Functional studies show reductions in the water permeability of lens fiber cell membranes in both homozygous and heterozygous mice (Varadaraj et al., 2005). However, even though the fiber cell membrane water permeability in heterozygous AQP0-null lenses is approximately half that of wild type (Varadaraj et al., 2005), gap junctional conductance and the gradients of intracellular hydrostatic pressure are similar between these lenses (Hall and Mathias, 2014). Because changes in gap junction coupling cause proportional changes in the intracellular hydrostatic pressure (Gao et al., 2011), the AQP0^+/−^ pressure data imply there are no effects on gap junctional coupling. This also implies that the changes in gap junctional plaque size detected in these lenses (Al-Ghoul et al., 2003) do not lead to decreased fiber cell-to-fiber cell coupling (at least in heterozygotes).

Changes in connexins and gap junctions in AQP0-mutant or -null mice might develop due to loss of normal interactions between AQP0 and connexins. Studies in chicken lenses suggest that AQP0 and Cx50 directly interact in differentiating fiber cells (Yu et al., 2005). In exogenous expression systems, co-expression of AQP0 increased the Cx50-mediated intercellular transfer of gap junction tracers by about 25% (Liu et al., 2011). Mutations in AQP0 that would alter such an interaction with Cx50 have been reported to affect transparency and gap junctional coupling in mouse lenses. Lenses from mice that are homozygous for the expression of a truncated form of AQP0 exhibit optical distortion and reduced transparency (Varadaraj et al., 2019). In addition, gap junctional coupling resistance is increased in the differentiating (1.6-fold) and mature (8-fold) fiber cells of these lenses, and lens hydrostatic pressure is increased by 1.5-fold in differentiating fibers and by 2-fold in the nucleus (Varadaraj et al., 2019). However, these changes in gap junction function occur without altering the levels of Cx46 or Cx50.

In addition, the cytoskeleton may influence the interaction between AQP0 and connexins. In the C57BL/6J strain (which contains beaded filaments), heterozygous AQP0-null mice have increased gap junctional coupling and decreased intracellular hydrostatic pressure in mature fibers as compared with wild type (Kumari et al., 2017), but, in the FVB strain (which lacks beaded filaments), these parameters are similar between heterozygous AQP0-null and wild-type lenses (Hall and Mathias, 2014). Although the fiber cell membrane permeability is similarly decreased in heterozygous AQP0-null of both strains compared to the respective wild types, the difference in gap junction coupling in heterozygous AQP0-null lenses may contribute to the reduced severity of the cataracts detected in the C57BL/6J strain compared to that in the FVB strain.

### Dystrophin deficient (*mdx* ^*3cv*^) mouse lenses

Dystrophin is a spectrin-related protein that contributes to a multi-protein complex that facilitates interactions between the cytoskeleton, the plasma membrane and the extracellular matrix. The lenses of dystrophin deficient (*mdx* ^*3cv*^) mice exhibit minor defects in transparency, developing punctate nuclear opacities in older animals (Karnam et al., 2021). The mutant mice show reductions of several cytoskeletal and membrane proteins, including Cx50; levels of Cx50 (detected by immunoblotting) and of Cx50-containing gap junctions (detected by immunofluorescence) are substantially reduced in the mutant mice. In contrast, immunoblots suggest that Cx46 levels are increased, but the distribution of Cx46 has not been studied.

### Eph/ephrin mutant lenses

The bidirectional Eph/ephrin signaling pathway is associated with both congenital and age-related cataracts in mice and humans. Lens fiber cells in mice lacking ephrin-A5 function appear rounded and irregular in cross-section, in contrast to their normal hexagonal appearance in wild-type lenses. Cataracts eventually develop in 87% of ephrin-A5 null mice (Cooper et al., 2008). EphA2^−/−^ lenses show very mild cortical or nuclear cataracts at weaning age (Cheng and Gong, 2011). Loss of EphA2 disrupts the nuclear compaction resulting in a small lens nucleus (Cheng et al., 2022). Although both EphA2 and ephrin-A5 knockout mice have abnormalities in the lens circulation and connexins (Cheng et al., 2021), there is no significant change in gap junction coupling in knockout lenses of either line. However, mature fiber cells of knockout lenses are hyperpolarized and display decreased membrane conductance compared to wild-type control lenses. The abundance of immunostained Cx50 (but not Cx46) plaques is reduced in peripheral lens fibers of homozygous EphA2-null mice.

### Deletion of β1-integrin in lens fiber cells

Conditional deletion of β1-integrin in lens fiber cells results in different effects on the lens: some lenses exhibit cataracts while others are clear but have refractive defects (Scheiblin et al., 2014). The transparent lenses have profound defects in fiber cell morphology associated with the loss of the F-actin network along lateral membranes. Interestingly, fiber cell gap junctional coupling is increased by 2-fold in these lenses. By immunofluorescence, the authors observed some qualitative changes in Cx46 and Cx50 gap junction plaque size and distribution, but with no convincing change in the levels of either Cx46 or Cx50. The increase in gap junctional coupling may explain the maintenance of lens transparency in the presence of profound structural abnormalities (Scheiblin et al., 2014).

### Selenite-induced nuclear cataracts

Injection of a single dose of sodium selenite into 10–14 day-old rats leads to the development of severe, bilateral nuclear cataracts within 5–6 days (Ostádalová et al., 1978). This model has been extensively used to examine development of cataracts and potential strategies to ameliorate their severity. Several events occur during cataract development in this model, including altered lens epithelial cell metabolism (and some cell death), calcium accumulation, crystallin precipitation, and disruption/loss of the cytoskeleton (reviewed by Shearer et al., 1997). The major pathologic processes are believed to be direct oxidative damage by the selenite and calpain-induced proteolysis activated by the elevated calcium levels. Analysis of membrane fractions from rats with selenite-induced cataracts showed a significant decrease in the more slowly migrating of two immunoreactive Cx46 bands (which likely represents phosphorylated Cx46) in the fraction from the cortex (but not other lens regions) (Fleschner, 2006). The reduction in the extent of Cx46 phosphorylation might result in alterations of gap junctional coupling, but this has not been investigated. Although proteolysis has been frequently implicated in the pathogenesis of cataracts in this model, the total abundance of full length Cx46 and Cx50 were not different between control and selenite-treated animals.

## Alterations of kinases and phosphatases may affect connexin phosphorylation and thereby fiber cell coupling

All of the lens connexins are phosphoproteins (Musil et al., 1990; TenBroek et al., 1992; Jiang et al., 1993; Berthoud et al., 1994; Gupta et al., 1994; Jiang et al., 1994; TenBroek et al., 1994; Berthoud et al., 1997; Shearer et al., 2008; Wang and Schey, 2009; Wang et al., 2013; Myers et al., 2018). Changes in phosphorylation of connexins (at different sites) can affect many aspects of the connexin life-cycle and channel function (Sáez et al., 2003; Solan and Lampe, 2005; Solan and Lampe, 2014; Leybaert et al., 2017).

### PKCγ knockout mouse lenses

Protein kinase Cγ is an isoform of the serine/threonine kinases that belongs to the diacylglycerol- and calcium-dependent classical subfamily of protein kinase C (PKC). PKCγ phosphorylates all lens connexins, and this phosphorylation can decrease gap junction intercellular communication. Deletion of PKCγ in mice does not induce cataracts. Lenses from PKCγ knockout mice show a 34% increase in gap junction coupling conductance in the differentiating fibers and an 82% increase in the mature fibers compared with wild-type lenses (Das et al., 2011). These changes are associated with a 150% increase in levels and a broader distribution of Cx43. Unlike wild-type lenses where Cx43 localization is largely restricted to epithelial cells, Cx43 is found in epithelial cells and cortical fiber cells in PKCγ knockout lenses. While expression of full-length Cx46 or Cx50 and their localization are not affected by deletion of PKCγ, serine phosphorylation of Cx46 is significantly decreased (Das et al., 2011). The PKCγ knockout lenses do not show changes in several other physiological parameters (including resting membrane potential, input resistance and pH gating of gap junction channels).

### Mitogen-activated protein kinase kinase 1

Transgenic mice that express a constitutively active mutant of Mitogen-activated protein kinase kinase 1 (MEK1) [MEK1(E)] under the control of the αA-crystallin promoter develop macrophthalmia and severe cataracts with major histological abnormalities of the lens that are followed by lens rupture (Gong et al., 2001). Interestingly, co-expression of a constitutively active MEK1 with Cx50 (but not Cx46) in pairs of *Xenopus* oocytes increases the Cx50-induced gap junctional conductance (Shakespeare et al., 2009). Studies of ionic homeostasis or components of the lens circulation (like fiber cell coupling conductance) are not possible in the MEK1(E) transgenic mice. However, studies of their lens epithelial cells have shown a large increase (2.6-fold) in the component of gap junctional coupling attributable to Cx50. Replacement of Cx50 in the MEK1(E) lenses with Cx46 leads to partial improvement of their phenotype (delayed lens rupture) (Shakespeare et al., 2009). These results suggest that maintenance of the normal interrelationship between Cx50 and MEK1 activity in the lens is essential for normal eye development, and lens transparency and function.

### PTEN knockout lenses

Patients with mutations in the human phosphatase and tensin homolog (PTEN) gene develop cataracts as part of the PTEN hamartoma tumor syndrome. Mice with a specific deletion of PTEN in the lens (PTEN knockout) also develop lens swelling, opacities and, eventually, rupture (Sellitto et al., 2013). PTEN knockout lenses exhibit several physiological abnormalities including an increase in intracellular hydrostatic pressure, increased intracellular sodium concentrations, and reduced Na^+^/K^+^-ATPase activity. Pharmacological inhibition of AKT (also known as protein kinase B) reduces lens pressure in these lenses and normalizes Na^+^/K^+^-ATPase activity in isolated lens cells. This suggests that PTEN regulates the lens circulation by modulating Na^+^/K^+^-ATPase activity (Sellito et al., 2013). It might be interesting to determine the functional state of lens gap junctions in these animals to clarify whether changes in gap junctional coupling contribute to the physiological abnormalities observed in these lenses. In exogenous expression systems, treatment of Cx50- (but not Cx46-) expressing cells with an AKT inhibitor decreases junctional conductance (Martinez et al., 2015). Based on these data and because PTEN is a negative regulator of the AKT/PKB signaling pathway, it would be expected that deletion of PTEN increases (instead of decreasing) gap junctional coupling.

## In some cataract models changes of fiber cell connexins and gap junction-mediated intercellular communication are uninvestigated, but may be anticipated

A large number of animal models that were developed using pharmacologic and genetic approaches have been used to study various changes that may be involved in cataractogenesis. In some of these models, reductions of fiber cell connexin levels or gap junction-mediated communication between fiber cells might be predicted, even though they have not yet been studied.

Recently, it was shown that mice with homozygous deletion of the gene encoding nuclear factor erythroid 2–related factor 2 (Nrf2, also known as Nfe2l2) develop significant cataracts late in life (11–15 months of age) at a higher frequency than wild-type mice of comparable ages (Rowan et al., 2021). Although the mechanism responsible for development of these cataracts has not been determined, it is possible that these mice have less protection against oxidative stress and therefore age faster. It would be interesting to examine aspects of the lens circulation, which may be affected by post-translational modifications and/or levels of pump-, transporter- and channel-forming proteins including the fiber cell connexins.

Atg5 (autophagy related 5) is involved in multiple cellular processes including ones that are relevant to lens biology like autophagic vesicle formation and mitochondrial quality control after oxidative damage. In mice with lens-specific disruption of the Atg5 gene (*Atg5* ^*flox/flox*^; MLR10-*Cre* mice), the lens undergoes normal morphogenesis and remains transparent through the first 5 months of age. However, by 6–9 months, lens opacities appear and progress with age. After 21 months, all *Atg5* ^*flox/flox*^;MLR10-*Cre* mice have severe bilateral cataracts, while a cataract was found in <10% of control mice, suggesting that Atg5 delays formation of age-related cataracts (Morishita et al., 2013). The age-related cataracts in the Atg5-deficient lens have been ascribed to a deficiency of constitutive autophagy, since these lenses have high density deposits in the cytoplasm of differentiating fiber cells and they accumulate insoluble oxidized proteins and crystallins, polyubiquitinated proteins and p62 (Morishita et al., 2013). Since ion and water physiology have not been investigated in these animals, it is reasonable to anticipate that the lens circulation will be disturbed, because many of the proteins that participate in the lens circulation (including Cx46 and Cx50) can be substrates for oxidation, ubiquitination and degradation by autophagy.

L-buthionine sulfoximine (L-BSO) has been used to induce cataracts that may model several aspects of oxidative damage. L-BSO is a specific inhibitor of the y-glutamylcysteine synthetase, the enzyme that catalyzes the first step of glutathione biosynthesis. Although it is relatively non-toxic in adult mice, repeated administrations of L-BSO to male suckling mice induces severe glutathione depletion and a variety of age-specific pathological changes in lenses. These mice develop dense cataracts, but they are free of other significant long-term effects and have excellent survival (Calvin et al., 1986). Fiber swelling that starts along the sutures is one of the very early events in the development of the BSO-induced mouse cataract (Calvin et al., 1991). This may reflect an osmotic effect with increased hydration of the lens. While the lenses of treated animals eventually exhibit various modifications of crystallin polypeptides and rapid deterioration of fiber cells, these changes are preceded by significant abnormalities in lens electrolyte composition including initial increase in Na^+^ and decrease in K^+^ in the pre-cataractous stage, followed by increased lens hydration, a pronounced increase in Ca^2+^, and further increase in Na^+^ and decrease in K^+^ (Calvin et al., 1991; Calvin et al., 1992a; Calvin et al., 1992b). These ionic changes suggest that the BSO-induced cataracts result at least in part from disruption of the lens circulation.

Mutations in the human *CHMP4B* gene underlie rare, inherited forms of early-onset lens opacities (Shiels et al., 2007a). This protein facilitates a diversity of membrane remodeling and scission processes, including endosomal sorting, exosome and ectosome release, plasma membrane repair, neurite pruning, nuclear envelope sealing, and autophagy. Therefore, it might be anticipated that mutations or deficiencies in this protein would affect plasma membrane lens proteins leading to abnormalities of lens fiber cells. Unfortunately, mice that are homozygous for a human cataract-associated mutation in *CHMP4B* (p.D129V) die during embryogenesis. However, conditional knockdown mice deficient in lens *CHMP4B* display severe lens dysmorphology or lens ablation (Zhou et al., 2019), supporting the hypothesis that *CHMP4B* mutations or deficiency affect membrane proteins, including the lens connexins.

## Conclusion

As reviewed above, the lens circulation of water and ions is altered or disrupted in a large number of mouse models that develop cataracts due to a variety of different initial causes. In many cases, connexin levels and the cell-to-cell communication that they facilitate are reduced. Therefore, we suggest that these may be central and critical components of cataractogenesis. The mechanisms leading to reductions of connexins and intercellular communication vary in different cataract models, and in many cases they remain unknown; they may be direct in some models and indirect in others.

While a substantial reduction in fiber cell communication likely contributes to cataractogenesis, it is not necessarily sufficient to cause cataracts by itself (at least not very rapidly), since aging mice have low levels of coupling even when most have clear lenses (Gao et al., 2013), heterozygous Cx46-null and Cx50-null mice have clear lenses (Gong et al., 1997; White et al., 1998; Rong et al., 2002), heterozygous Cx46fs380 mice have very low levels of coupling long before they develop cataracts (Minogue et al., 2017), and GPX-1 knockout lenses show decreased coupling conductance at 2 months when they are clear (Wang et al., 2009).

Taking together these studies suggest that a “normal” level/range of connexin abundance and of coupling must be maintained in the lens to avoid opacification. A 50% reduction in connexin levels is adequate in the heterozygous connexin null mice (Gong et al., 2001). But, an increase in coupling (as may be the case in the MEK1 model) or a gross overexpression of Cx50 (as in the Cx50 transgenic mice) can be detrimental (Chung et al., 2007; Shakespeare et al., 2009).

Finally, some cataract models develop cataracts without apparent changes in connexins or fiber cell gap junctions. A severe abnormality of a single major lens component (like some of the crystallins) or disruption of the lens structure (like modified fiber cell shape or altered layering of fiber cells) can cause cataract.
